# Uncontrolled Manifold Reference Feedback Control of Multi-Joint Robot Arms

**DOI:** 10.3389/fncom.2016.00069

**Published:** 2016-07-12

**Authors:** Shunta Togo, Takahiro Kagawa, Yoji Uno

**Affiliations:** ^1^Cognitive Mechanisms Laboratories, Advanced Telecommunications Research Institute InternationalKyoto, Japan; ^2^Japan Society for the Promotion of ScienceTokyo, Japan; ^3^Graduate School of Engineering, Nagoya UniversityNagoya, Japan

**Keywords:** joint coordination, uncontrolled manifold analysis, synergy, feedback control, redundant arm, tracking task

## Abstract

The brain must coordinate with redundant bodies to perform motion tasks. The aim of the present study is to propose a novel control model that predicts the characteristics of human joint coordination at a behavioral level. To evaluate the joint coordination, an uncontrolled manifold (UCM) analysis that focuses on the trial-to-trial variance of joints has been proposed. The UCM is a nonlinear manifold associated with redundant kinematics. In this study, we directly applied the notion of the UCM to our proposed control model called the “UCM reference feedback control.” To simplify the problem, the present study considered how the redundant joints were controlled to regulate a given target hand position. We considered a conventional method that pre-determined a unique target joint trajectory by inverse kinematics or any other optimization method. In contrast, our proposed control method generates a UCM as a control target at each time step. The target UCM is a subspace of joint angles whose variability does not affect the hand position. The joint combination in the target UCM is then selected so as to minimize the cost function, which consisted of the joint torque and torque change. To examine whether the proposed method could reproduce human-like joint coordination, we conducted simulation and measurement experiments. In the simulation experiments, a three-link arm with a shoulder, elbow, and wrist regulates a one-dimensional target of a hand through proposed method. In the measurement experiments, subjects performed a one-dimensional target-tracking task. The kinematics, dynamics, and joint coordination were quantitatively compared with the simulation data of the proposed method. As a result, the UCM reference feedback control could quantitatively reproduce the difference of the mean value for the end hand position between the initial postures, the peaks of the bell-shape tangential hand velocity, the sum of the squared torque, the mean value for the torque change, the variance components, and the index of synergy as well as the human subjects. We concluded that UCM reference feedback control can reproduce human-like joint coordination. The inference for motor control of the human central nervous system based on the proposed method was discussed.

## Introduction

There are redundant relationships underlying the motor control of the human body. For instance, a simple voluntary reaching movement involves a redundant hand trajectory, redundant joint angles, and redundant muscles. We focused on the redundancy between the hand position as a single target and the greater number of degrees of freedom (DOFs) of the joints.

To approach the above redundancy problems, Bernstein (1967) pointed out that “the human controls his/her redundant DOF of body by using joint coordination.” Specifically, the joint coordination is a control strategy in which redundant elements (motor elements) are varied without affecting the variable that must be controlled to achieve the task (performance variable). In other words, the human central nervous system (CNS) facilitates families of solutions equally able to solve the task as described by Latash's Principle of Abundance (Latash, 2012). Therefore, joint coordination can flexibly stabilize the performance variables. To quantify the joint coordination of human movements, uncontrolled manifold (UCM) analysis has been proposed (Scholz and Schöner, 1999). UCM analysis divides the trial-to-trial variance of redundant motor elements into two orthogonal components: a UCM component that does not affect the performance variable and an ORT (orthogonal) component that directly affects the performance variable. If a particular performance variable is controlled by the coordination of motor elements, the UCM component is greater than the ORT component. UCM analysis has been used to investigate many types of human tasks including the reaching movement (Domkin et al., 2002, 2005; Tseng et al., 2002, 2003; Yang et al., 2007), and has suggested a coordinated structure for voluntary human movement (Latash et al., 2002, 2007; Latash, 2010).

Within the framework of the UCM, the task can be achieved by only controlling the ORT component. This concept in which only the task-relevant elements can be controlled was also mentioned as the “minimal intervention principle” by Todorov and Jordan (2002a). They introduced a traditional optimal feedback control framework to achieve the principle (Todorov and Jordan, 2002b). In the framework of optimal feedback control, however, there is neither a control model explicitly using the UCM nor an evaluation of the joint coordination. Moreover, to apply the framework to a nonlinear system, which is the nature of the human body, a complicated algorithm is needed (Todorov et al., 2005; Li and Todorov, 2007; Liu and Todorov, 2009).

In this paper, we propose a novel control method of human redundant joints, which achieves the “minimal intervention principle” using the UCM directly. The main purpose of the present study is to confirm that our proposed method can generate arm movements with human-like joint coordination at a behavioral level. Specifically, we consider a one-dimensional target-tracking task that is more redundant than the traditional two-point reaching movement. In this task, a target of the hand in the lateral direction is provided. As a conventional method, we considered the method that pre-determined a unique target joint trajectory by inverse kinematics or any other optimization method. The controller tracked the pre-determined unique target trajectory. Standard inverse-kinematics approaches cannot implement motor variability in the joint space without violating the task space constraint, which are given by the tracking of an end-effector pose. Therefore, the conventional method does not model appropriately the variability of joint angles. In contrast, our proposed method generated the target UCM from the given target position of the hand, and selected the optimal joint combination in the target UCM at each time step.

The proposed method does not generate optimal and unique target joint trajectory. Instead, the optimal joint combination in the target UCM is determined each time step. During arm movements, an arm posture is disturbed by a noise of motor command and an external perturbation. By selecting the optimal joint combination in the target UCM, the variability of joint due to the noise and perturbation is minimally corrected. Therefore, the variability of the joint along the UCM is allowed and results in joint coordination. Moreover, the controller can smoothly and effortlessly respond to the perturbation by allowing variability in the target UCM. We refer to the proposed method as “UCM reference feedback control.” To check whether our proposed method can generate human-like joint coordination, we conducted measurement experiments of human subjects performing a one-dimensional target-tracking task. We quantitatively compared the performances of the arm movements and joint coordination quantified using the UCM analysis in simulation experiments with those from the measurement experiments.

## Materials and methods

### UCM reference feedback control

#### One-dimensional target-tracking task

**Figure 1:**
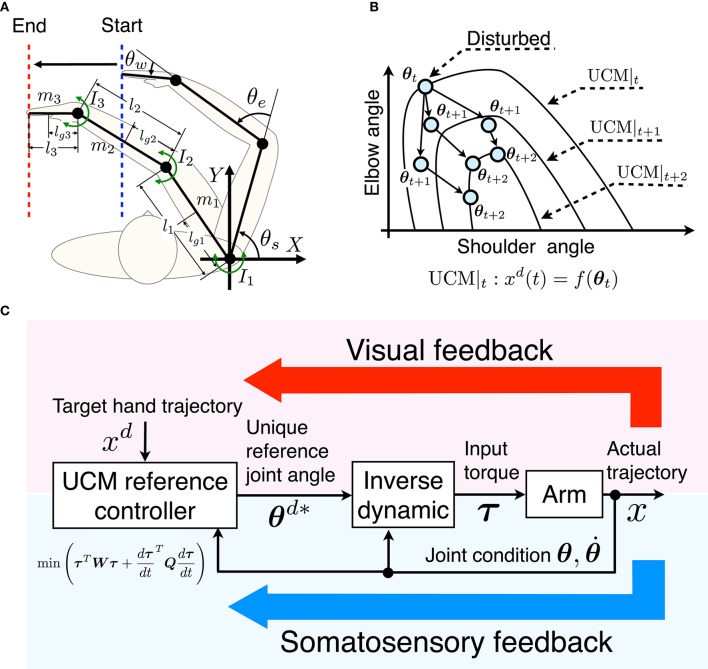
**Concept of UCM reference feedback control**. **(A)** Schematic diagram of the task coordination and joint angles in the horizontal plane. For the one-dimensional target-tracking task, the hand is moved from the start position to the end position tracking the target hand position along the *X*-axis while variability along the *Y*-axis is allowed. **(B)** Schema of UCM reference feedback control. UCM_*t*_ denotes the target UCM at time *t*. The configuration of the joint angles is disturbed at time *t*, and deviates from the target UCM at time *t* + 1. Through the UCM reference feedback control method, the disturbed joint angles converge to the target UCM at time *t* + 2. **(C)** Block diagram of UCM reference feedback control. The UCM reference feedback controller is given the target hand trajectory *x*^*d*^, and calculates the unique reference joint angles **θ**^d*^ within the target UCM so as to minimize the cost function. Then, the inverse dynamics of the arm generates the input torque **τ** using the current joint condition **θ** and $\overset{\cdot }{\theta }$ and reference joint angles **θ**^d*^. The upper (pseudo) loop represents the visual feedback loop while the lower loop represents the somatosensory feedback.

In this study, the common controlled object in the simulation and measurement experiments is a three-link arm consisting of an upper arm, forearm, and hand. The common task is a tracking task of the one-dimensional target of the hand in the horizontal plane (Figure 1A). Thus, the joint angles and hand position correspond to the motor elements and the performance variable in the UCM concept. Only the lateral position of the target is considered so that the relationship between the target and motor elements is considered redundant. The task coordinates (*X*- and *Y*-axes) and joint angles (θ_*s*_, θ_*e*_, and θ_*w*_) are defined in Figure 1A.

The one-dimensional target of the hand is provided for in the medial–lateral direction (*X*-axis), and is calculated according to the minimum jerk criterion of the start and end velocities equaling zero (Flash and Hogan, 1985). Meanwhile, the hand is allowed to take any position in the anterior–posterior direction (*Y*-axis). The origin is the center of gyration of the shoulder. The arm movements are considered for two initial positions: a far position (θ_*s*_ = π/3 rad, θ_*e*_ = π/3, and θ_*w*_ = π/6 rad) and a near position (θ_*s*_ = π/4 rad, θ_*e*_ = π/2 rad, and θ_*w*_ = π/6 rad). These conditions indicate that initial hand position is either far or near from the shoulder. From these initial positions, the hand moves 0.3 m in 5 s. This movement is slower than an ordinary reaching movement because the present study considers accurate target-tracking movements of the human.

#### Brief description of UCM reference feedback control

UCM is a manifold that can be defined when the motor elements have redundant DOF(s) with respect to the performance variable. In the tracking task, any value of a joint angle within the UCM results in the same value of the hand position. Thus, the combination of the joint angles within the UCM accomplishes the task. To simplify the explanation of the UCM, we give an example of the UCM in the target-tracking task with a two-link arm consisting of a shoulder and elbow, and a one-dimensional target trajectory of the hand as shown in Figure 1B. The horizontal and vertical axes denote the shoulder and elbow angles (motor elements), and UCM|_*t*_ denotes the target UCM at time *t*. At each time step, our proposed method first generates and refers the target UCM, then, determines optimal joint combination in the target UCM, finally, input torque is calculated by inverse dynamics.

UCM reference feedback control first generates the target UCM (UCM|_*t*_) for a given target position *x*^*d*^(*t*). Next, a unique joint combination in the target UCM (the target joint combination) is selected so as to minimize a cost function for the current joint combination. Here we consider a cost function consisting of torque and the torque change terms that encourages small and smooth joint torques so as to represent a human's smooth and effortless movement. For instance, when the joint combination at time *t* (θ_*t*_) is disturbed and the joint combination θ_*t*__+1_ turns away from the target (Figure 1B), the optimal target joint combination in the UCM at *t* + 2 (θ_*t*__+2_) is selected so as to minimize the torque and torque change.

Finally, the input torque is calculated to track the optimal target joint combination using a current state feedback and inverse dynamics of the arm. Since the optimal joint combination depends on the current state, the target joint combinations are different trial by trial. Therefore, the UCM reference feedback control can achieve the task while permitting variance of the joints in the UCM. Such a mechanism that allows task-irrelevant variability of joints generates the joint coordination. The block diagram of our proposed method is shown in Figure 1C. The upper (pseudo) loop and lower loop correspond to the visual feedback and somatosensory feedback, respectively. To simplify the problem, we assume that there is no time delay or noise in the somatosensory feedback system.

#### Algorithm for UCM reference feedback control

An algorithm for the implementation of the UCM reference feedback control is explained. We now consider the one-dimensional target-tracking task in a horizontal plane with a three-link arm (Figure 1A). The equation of motion for the three-link arm is:

(1) $$\begin{array}{l} M\left(\theta \right)\ddot{\theta }+V\left(\dot{\theta },\theta \right)+D\dot{\theta }=\tau \end{array}$$

where $M\ddot{\theta }$ is an inertial term, ***V*** is a centripetal and Coriolis term and $D\dot{\theta }$ is a viscous term. The state variables are the joint angles and angular velocities.

A relationship between the joint angles (motor elements) and the hand position (performance variable) is represented by the following kinematics equation (UCM):

(2) $$\begin{array}{l} x^{d}=l_{1}\cos\left(\theta_{s}\right)+l_{2}\cos\left(\theta_{s}+\theta_{e}\right)+l_{3}\cos\left(\theta_{s}+\theta_{e}+\theta_{w}\right) \end{array}$$

where θ_*s*_, θ_*e*_, and θ_*w*_ are subject to Equation (2) when generating the UCM. A given target hand position is represented by *x*^*d*^. Hence, the update of *x*^*d*^ changes the UCM in a step by step manner. The target joint combination subject to the UCM as represented by Equation (2) is determined so as to minimize a cost function consisting of the joint torque and torque change terms. Thus, we consider the cost function to select the target joint combination:

(3) $$\begin{array}{l} E=\tau^{T}\text{W}\tau +{\frac{d\tau }{dt}}^{T}\text{Q}\frac{d\tau }{dt} \end{array}$$

where ***W*** = diag[*W*_*s*_, *W*_*e*_, *W*_*w*_] and ***Q*** = diag[*Q*_*s*_, *Q*_*e*_, *Q*_*w*_] are weighted matrices.

Next, we explain how to solve an optimization problem that minimizes Equation (3) with the constraint condition of Equations (1) and (2). The input torque and input torque change for the cost function (3) are calculated from the motion equation of the arm (1) as follows. To represent the torque and torque change by θ_*s*_, θ_*e*_, and θ_*w*_ in the UCM Equation (2) as variables, Equation (1) is approximated using the Euler method. To represent a relationship between the input torque (τ_*t*_) at *t* and a joint combination θ_*t*__+2_ at *t* + 2, Equation (1) is approximated as:

(4) $$\begin{array}{l} \theta_{t+1}=\theta_{t}+\Delta t{\dot{\theta }}_{t} \end{array}$$

(5) $$\begin{array}{l} {\dot{\theta }}_{t+1}={\dot{\theta }}_{t}+\Delta tM^{-1}(\tau_{t}-D{\dot{\theta }}_{t}-V) \end{array}$$

(6) $$\begin{array}{l} \theta_{t+2}=\theta_{t+1}+\Delta t{\dot{\theta }}_{t+1} \end{array}$$

where Δ*t* indicates the sampling duration in the Euler method. Substituting Equations (4) and (5) into Equation (6), a relationship between the target joint combination two steps latter $\theta_{t+2}^{d}$ and the current input torque is obtained:

(7) $$\begin{array}{l} \theta_{t+2}^{d}=\theta_{t}+2\Delta t{\dot{\theta }}_{t}+{\left(\Delta t\right)}^{2}M^{-1}\left(\tau_{t}-D{\dot{\theta }}_{t}-V\right) \end{array}$$

To simplify a calculation, the joint combinations **θ**^†^_*t*__+2_ and **θ**^‡^_*t*__+2_, which are generated by setting τ_*t*_ = 0 and τ_*t*_*=* τ_*t*__−1_, are calculated by using forward dynamics in Equation (7) according to:

(8) $$\begin{array}{l} \theta_{t+2}^{†}=\theta_{t}+2\Delta t{\dot{\theta }}_{t}+{\left(\Delta t\right)}^{2}M^{-1}\left(0-D{\dot{\theta }}_{t}-V\right) \end{array}$$

(9) $$\begin{array}{l} \theta_{t+2}^{‡}=\theta_{t}+2\Delta t{\dot{\theta }}_{t}+{\left(\Delta t\right)}^{2}M^{-1}\left(\tau_{t-1}-D{\dot{\theta }}_{t}-V\right) \end{array}$$

Equations (8) and (9) are used for calculation of input torque and input torque change. Subtracting Equations (8) and (9) from Equation (7) and transforming the result, the input torque τ_*t*_ and input torque change $\dot{\tau }=(\tau_{t}-\tau_{t-1})/\Delta t$ are obtained as:

(10) $$\begin{array}{l} \tau_{t}=M\frac{\theta_{t+2}^{d}-\theta_{t+2}^{†}}{{\left(\Delta t\right)}^{2}} \end{array}$$

(11) $$\begin{array}{l} \frac{\tau_{t}-\tau_{t-1}}{\Delta t}=M\frac{\theta_{t+2}^{d}-\theta_{t+2}^{‡}}{{\left(\Delta t\right)}^{3}} \end{array}$$

Therefore, the cost function in Equation (3) can be represented as a function of the target joint combination. By substituting Equation 2 for the UCM into the cost function, an optimal solution is obtained for the joint combination in the target UCM thereby guaranteeing the optimal joint combination that corresponds to the target hand position. To simplify the calculation, we use the UCM as linearized by the Jacobian:

(12) $$\begin{array}{l} x^{d}-x^{†}=J\left(\theta^{†}\right)\left(\theta_{t+2}^{d}-\theta^{†}\right) \end{array}$$

(13) $$\begin{array}{l} x^{d}-x^{‡}=J\left(\theta^{‡}\right)\left(\theta_{t+2}^{d}-\theta^{‡}\right) \end{array}$$

where ***J*** is the Jacobian matrix of Equation (2), and *x*^†^ and *x*^‡^ are the hand positions corresponding to **θ**^†^ and **θ**^‡^, respectively. Equations (12) and (13), which are linearized around **θ**^†^_*t*__+2_ and θ^‡^_*t*__+2_, are substituted into the expression for the torque in Equation (10) and the torque change in Equation (11), respectively. $\theta {\;}_{s}^{d}$ eliminated by substitution. Here, we assume that **θ**^†^_*t*__+2_ and **θ**^‡^_*t*__+2_ are near the target UCM two steps latter. To calculate the cost function (3), the other variables (i.e., the inertial term ***M***, the target hand position *x*^*d*^, **θ**^†^_*t*__+2_, and **θ**^‡^_*t*__+2_ obtained by the forward dynamics) are required. Combining them into a constant term ***A*** (details are shown in **Appendix A**), the cost function can be represented as a function of $\theta_{e}^{d}$ and $\theta_{w}^{d}$:

(14) $$\begin{array}{l} E=A_{1}{\left(\theta_{e}^{d}\right)}^{2}+A_{2}\theta_{e}^{d}+A_{3}\theta_{e}^{d}\theta_{w}^{d}+A_{4}\theta_{w}^{d}+A_{5}{\left(\theta_{w}^{d}\right)}^{2}+A_{6} \end{array}$$

To calculate the extreme values for $\theta_{e}^{d*}$ and $\theta_{w}^{d*}$, Equation (14) is partially differentiated with respect to $\theta_{e}^{d}$ and $\theta_{w}^{d}$. The value $\theta_{s}^{d*}$ is calculated by substituting $\theta_{e}^{d*}$ and $\theta_{w}^{d*}$ for which the cost function (3) takes a minimal value into Equation (2); therefore, that the target joint combination is in the target UCM. From the above calculations, the optimal solution can be analytically obtained, and the target joint combination can thus be calculated without any iteration. Then, substituting the target joint combination **θ**^d*^ into Equation (7) and solving the equation for the inverse dynamics, the actual input torque τ_*t*_ can be calculated as:

(15) $$\begin{array}{l} \tau_{t}=M\frac{\theta_{t+2}^{d*}-\theta_{t}-2\Delta t{\dot{\theta }}_{t}}{{\left(\Delta t\right)}^{2}}+D\dot{\theta }+V \end{array}$$

The above algorithm is implemented for the three-dimensional motor elements and the one-dimensional performance variable. In general, even if the motor elements are *n*-dimensional and the performance variable is *m*-dimensional (*m* < *n*), our proposed method can be directly applied. It should be noted that a more complex equational representation of the analytical solution is required for a higher dimensional system.

### Simulation experiment

A computer simulation of the UCM reference feedback control was performed for the one-dimensional target-tracking task. The physical parameters of a three-link arm were calculated using anthropometric data (Winter, 2004). The values given in Table 1 were calculated from the length of the body segments and the body weight of a typical subject in the measurement experiment. The value of the viscous matrix ***D*** is given in Table 2. The initial posture is given by the measurement data of the typical subject. The sampling duration was 0.0083 s (at 120 Hz, which corresponds to the sampling rate of the measurement experiments). The fourth-order Runge–Kutta method was used for the integration of the motion equation of the arm. Since the wrist torque should be smaller than shoulder and elbow torques in the three-link arm reaching, we specified *W*_*s*_ = 1/$I_{1}^{2}$, *W*_*e*_ = 1/$I_{2}^{2}$, and *W*_*w*_ = 1/$I_{3}^{2}$ as the weighted terms in the cost function in Equation (3). According to the minimum torque change criterion that can reproduce human reaching movements (Uno et al., 1989), the weight of the torque change term was a unit matrix (i.e., *Q*_*s*_ = *Q*_*e*_ = *Q*_*w*_ = 1).

**Table 1:** **Arm parameters**.

	**Link1**	**Link2**	**Link3**
*m_i_* kg	1.88	1.07	0.40
*l_i_* m	0.24	0.25	0.17
*l_gi_* m	0.10	0.11	0.13
*I_i_* kgm^2^	3.05 × 10^−2^	1.88 × 10^−2^	0.88 × 10^−2^

*m_i_, l_i_, l_gi_, and I_i_ correspond to the physical parameters shown in Figure 1A (i = 1, 2, 3)*.

**Table 2:** **Viscous matrix in the simulation**.

**Body parts**	**Shoulder**	**Elbow**	**Wrist**
**JOINT VISCOSITY D NMS/RAD**			
Shoulder	1.5	0.5	0
Elbow	0.5	1.0	0
Wrist	0	0	0.4

*Diagonal elements indicate the joint viscosities of monoarticular muscles and off-diagonal elements indicate those of biarticular muscles. Values of zero indicate that there are no biarticular muscles between the shoulder and wrist, and between the elbow and wrist*.

To reproduce the variability of the human arm movement, noise was added to the three variables in Figure 1C. The added noise is considered reasonable for biological motor systems. Additionally, the simulation was run for 100 trials for both the far and near initial positions. The first of the three variables is the perceived target hand position *x*^*d*^. We assumed uncertainty as caused by visual localization in visual perception:

(16) $$\begin{array}{l} x_{in}^{d}=x^{d}+e_{x}v^{d}N\left(0,1\right), \end{array}$$

where *N*(0, 1) is a normal distribution (mean 0, variance 1), *x*^*d*^ is the given target hand position, $x_{in}^{d}$ is the perceived target position used in UCM reference feedback control, *v*^*d*^ is the target velocity and *e*_*x*_ is the noise amplitude. We assumed that the perception of the moving target was more uncertain than that of a static target. Therefore, the visual perception noise was proportional to the target velocity. The second variable was the optimal joint angle **θ**^d*^. Assuming that there was uncertainty in acquiring the target UCM, we used

(17) $$\begin{array}{l} \theta_{in}^{d*}=\theta^{d*}+e_{\theta }N\left(0,1\right), \end{array}$$

where $\theta_{in}^{d*}$ is the target joint combination substituted into the inverse dynamics of the arm (Equation 15) and *e*_θ_ is the noise amplitude.

The third variable is the input torque τ_*t*_. Harris and Wolpert (1998) suggested that the hand trajectory in a reaching movement varied owing to signal-dependent noise (SDN) in the motor command. In this study, we assumed SDN in joint torque:

(18) $$\begin{array}{l} \tau_{in}=\left(U+e_{\tau }N\left(0,1\right)\right)\tau_{t}, \end{array}$$

where ***U*** is an identity matrix, **τ**_*t*_ is the joint torque generated by the inverse dynamics (Equation 15), **τ**_*in*_ is the actual joint torque inputted to the arm and *e*_τ_ is the noise amplitude. The noise amplitudes *e*_*x*_, *e*_θ_, and *e*_τ_ were heuristically determined so as to quantitatively fit the mean value data across all subjects, especially the total variance as shown in Figure S1 (**Appendix B**). The profile of the sum of the squared torque is shown in **Figure 6**. (specifically, *e*_*x*_ = 1.5 × 10^−5^, *e*_θ_ = 1.0 × 10^−6^, and *e*_τ_ = 0.22). We evaluated the initial and end positions, sum of the squared tracking error (i.e., the difference between the hand and target position), the tangential hand velocity, input joint torque and torque change in this simulation. Additionally, joint coordination was quantitatively evaluated through UCM analysis. We calculated the mean value data for the above kinematics and dynamics across 100 trials and compared them to mean value data across all subjects in the measurement experiments by one-sample *t*-test.

### Measurement experiment

Eight healthy right-handed male subjects participated in the measurement experiments. The experiments were approved by the Nagoya University Ethical Review Board. All subjects were provided with explanations regarding the experimental procedure and gave their written informed consent.

**Figure 2:**
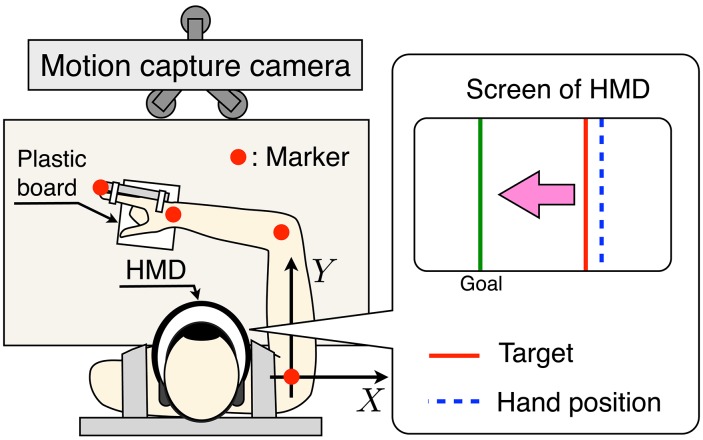
**Schematic diagram of the measurement experiment setup**. Subjects perform a one-dimensional target-tracking task while sitting in a chair and wearing a seatbelt. A head-mounted display (HMD) is secured to the individual's head. Markers are placed on the arm segments identified in Figure 2. The one-dimensional hand position of the subject and the target hand position are shown by screen of the HMD.

We used a 3D position measurement system (OPTOTRAK CERTUS, Northern Digital Inc.) to record the kinematics at 120 Hz. Infrared-ray markers with a diameter of 7 mm were placed on four anatomical landmarks of a subject's arm: the center of gyration of the shoulder, the elbow, the wrist, and the tip of the index finger (Figure 2). The index finger was fixed at an extended position so that the length of the hand (*l*_3_) was sufficiently long. A plastic board that was easy to slide on a desk was placed under the hand. Subjects secured a head-mounted display (HMD) (HMZ-T1, SONY Inc.) to their heads so as to obtain their hand positions and the target hand position in the medial–lateral direction. The refresh rate of the screen of the HMD was 60 Hz.

Subjects performed the one-dimensional target-tracking task while sitting on a chair and wearing a seatbelt. The far position task and near position task were each performed for 100 trials. Thus, the subjects performed the one-dimensional target-tracking task for 200 trials. To confirm that there was no effect of the task order, four subjects performed the far position task first, and the other four subjects performed the near position task first. When the subjects felt fatigued, they took a short break arbitrarily. To adjust the initial positions for all trials, the subjects moved their elbow, wrist, and fingertip for the target of the initial posture with accuracy of ±2 mm using information from the HMD.

In the initial position control phase, the lengths of all links were calculated from the kinematics data, and the target of the initial posture was calculated with the kinematics equation so as to meet the task condition (far or near). After this phase, a countdown of 3 s was displayed on the HMD. The one-dimensional hand position at the movement end, target hand position and subject's hand position were then displayed as shown in Figure 2. Subjects performed the one-dimensional target-tracking task only using the information on the HMD. At the end of each trial, subjects were given feedback of the sum of the tracking error. They were instructed to track the target hand position so as to reduce the tracking error. In addition, they were asked to keep their elbow horizontal so as to avoid friction with the desk. To eliminate outlier or failure trials, we applied the following two screening processes. First, we eliminated the trials in which the value of the squared sum of the tracking error was larger than the mean value plus 2 standard deviations. Second, the trials in which the value of the mean sum of the squared torque change was larger than the mean value plus 1 standard deviation were rejected. Finally, 79.3 ± 13.3 trials were analyzed.

The position data were filtered with a second-order Butterworth low-pass filter with a 10-Hz cutoff frequency. We obtained the start and end positions, the tangential velocity of the hand and the joint torque from these position data. The tangential velocity of the hand was obtained by calculating the differential of the measured position and then filtered with a second-order Butterworth low-pass filter with a 10 Hz cutoff frequency. Using the same method, we obtained the joint angle, angular velocity, and angular acceleration from the measured position, and obtained the joint torque from the inverse dynamics of the arm in Equation (1) using the same viscous matrix as that in the simulation experiment (Table 2).

#### UCM analysis

The joint coordination was evaluated through the following UCM analysis. The UCM is the nonlinear manifold associated with the redundant kinematics, and is locally linearized by the null space of the Jacobian ***J*** between the hand velocity in the *X*-direction and the joint velocity.

The variances parallel to the UCM and orthogonal to the UCM are called the UCM component (*V*_UCM_) and ORT component (*V*_ORT_), respectively. These variance components can efficiently be computed from the covariance matrix C of the observed joint angles (Yen and Chang, 2009):

(19) $$\begin{array}{l} V_{\text{UCM}}=\frac{\text{trace}\left(\text{null}{\left(J\right)}^{T}C\text{null}\left(J\right)\right)}{n-d} \end{array}$$

(20) $$\begin{array}{l} V_{\text{ORT}}=\frac{\text{trace}({\left(JJ^{T}\right)}^{-1}JCJ^{T})}{d} \end{array}$$

(21) $$\begin{array}{l} V_{\text{TOT}}=\frac{\text{trace}\left(C\right)}{n} \end{array}$$

where *V*_TOT_ is the total variance. These variance components are normalized by the dimensions of the joint combination *n* (*n* = 3), the dimensions of the hand position *d* (*d* = 1), and the dimensions of the null space *n*–*d*. To evaluate the degree of joint coordination, we define the index of synergy σ^*^ (Verrel, 2010) as:

$$\begin{array}{l} \sigma^{*}=\log\left(\frac{\sigma +n∕d}{n∕\left(n-d\right)\;-\sigma }\right) \end{array}$$

(22) $$\begin{array}{l} \sigma =\frac{V_{\text{UCM}}-V_{\text{ORT}}}{V_{\text{TOT}}} \end{array}$$

when *V*_UCM_ = *V*_ORT_ then the joints are not coordinated. Thus, σ^*^ > log((*n*/*d*)/(*n*/(*n–d*))) = 0.69 means that the joints are varied parallel to the UCM, which implies a synergetic stabilization of the performance variable (Latash, 2010).

## Results

We confirmed that there was no effect of task order on the performance and characteristics of the movements.

### Kinematic and dynamic properties

**Figure 3:**
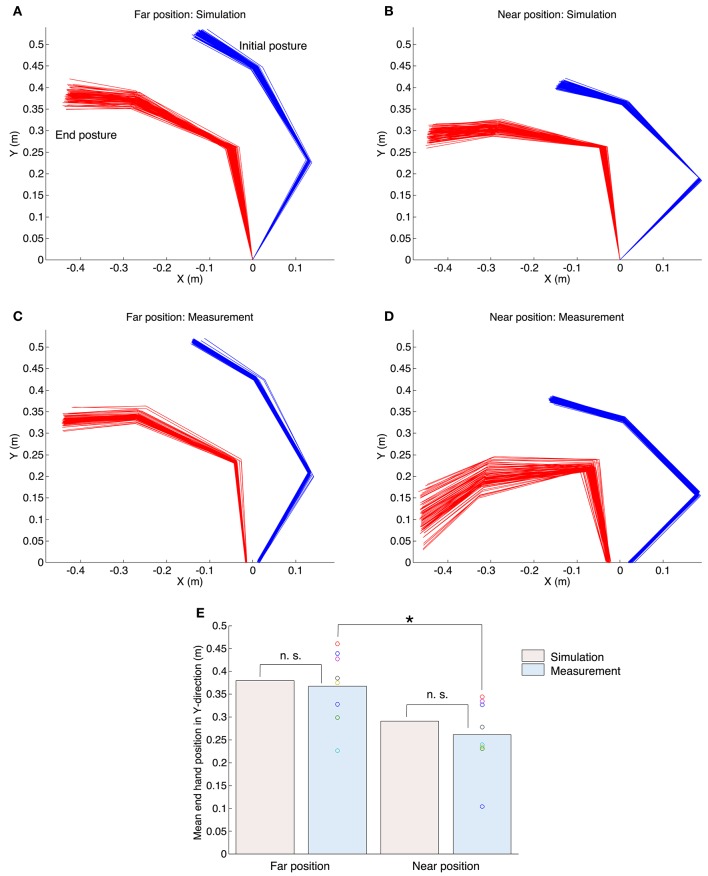
**Initial and end postures of the arm in the simulation and measurement experiments**. The upper left **(A,C)** and upper right **(B,D)** graphs correspond to the far position task and near position task. The horizontal axis and vertical axis denote the medial–lateral direction and anterior–posterior directions. The blue lines denote the initial posture and the red lines denote the end postures. The origin is the center of gyration of the shoulder. **(E)** The mean value of the end hand position in the *Y*-direction for all experiments. The n. s. indicates not significant (one-sample *t*-test), and the asterisk denotes a significant difference (paired *t*-test, *P* < 0.05).

Figure 3 shows the start and end arm postures. The upper (Figures 3A,B) and middle (Figures 3C,D) figures indicate the results of the simulation and the measurement experiments. In both experiments, hand positions at the movement end were more varied in the anterior–posterior direction (*Y*-direction), and the variance of joint angles did not affect task achievement. Hand positions in the near position task were nearer the trunk at the movement end than those in the far position task. Figure 3E shows the mean hand position at the movement end in the Y-direction for all experiments. Our proposed method generated quantitatively similar hand positions to those of all subjects. Statistically, a one-sample *t*-test between the simulation and measurement results showed no significant difference [the far position task: t_(7)_ = −0.45, *P* = 0.67; the near position task: t_(7)_ = −1.06, *P* = 0.33]. Moreover, a paired *t*-test demonstrated that the measured hand positions in the near position task were significantly nearer than those in the far position task [t_(7)_ = 4.50, *P* = 0.0028 < 0.05], and the proposed method could generate same tendency (the far position task: 0.38 m; the near position task: 0.29 m).

**Figure 4:**
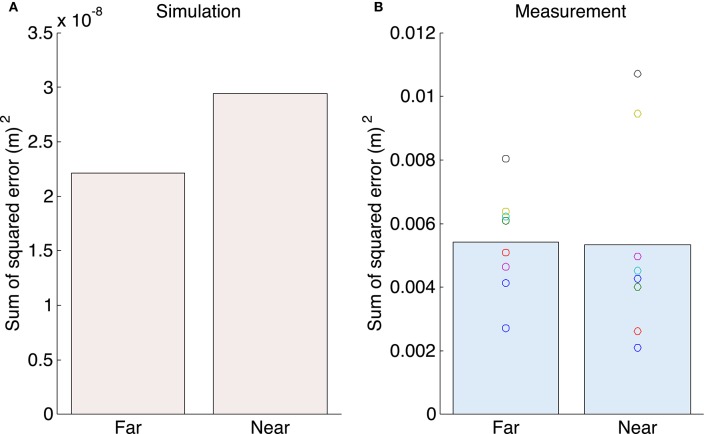
**The sum of the squared tracking error. (A)** The mean value for the tracking error for the simulation experiments. **(B)** The mean value for the tracking error for all subjects for the measurement experiments.

Figure 4 shows the sum of the squared tracking error. Our proposed method could more accurately track the target than the human subjects. A one-sample *t*-test between the simulation and measurement results showed significant difference between the tracking error of the simulation and measurement experiments [the far position task: *t*_(7)_ = 9.39, *P* = 3.24 × 10^−5^ < 0.05; the near position task: *t*_(7)_ = 4.85, *P* = 0.0019 < 0.05].

**Figure 5:**
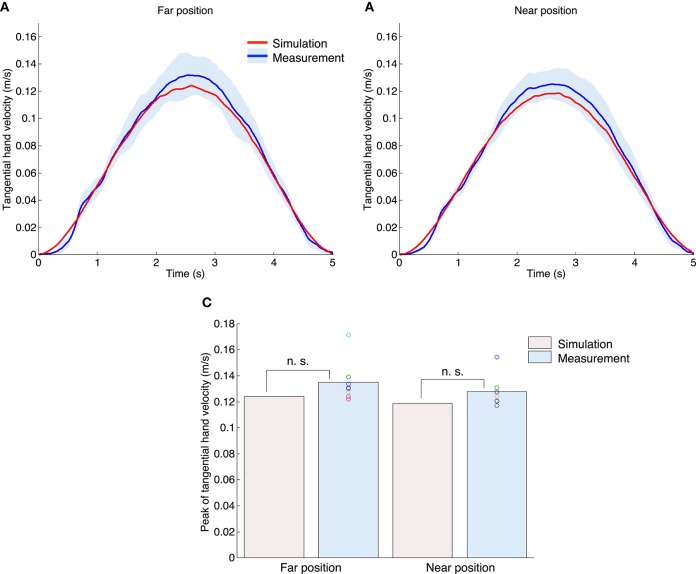
**Tangential hand velocity for both the simulation and measurement experiments**. The upper left **(A)** and right **(B)** graphs denote the far position and near position tasks. The red and blue lines indicate the mean value profiles for the tangential hand velocity across all trials for the simulation experiments, and across all subjects for the measurement experiments. The blue area denotes the standard deviation across all subjects. **(C)** Peak of tangential hand velocity of all experiments. The n. s. indicates not significant (one-sample *t*-test), and the asterisk denotes a significant difference (paired *t*-test, *P* < 0.05).

Figures 5A,B show the profiles of tangential velocity of the hand. The red and blue solid lines indicate the results of simulation and measurement experiments. The blue area denotes standard deviation across all subjects. The target hand trajectory in the one-dimensional target-tracking task had a bell-shaped and smooth velocity profile with a peak at the middle of the movement duration (2.5 s). In both the simulation and measurement experiments, the profiles of the tangential velocity of the hand were also bell-shaped. Figure 5C shows the peak of the tangential hand velocity for all experiments. A one-sample *t*-test between the simulation and measurement results indicated that our proposed method could generate a similar peak compared to the measurement experiments [the far position task: *t*_(7)_ = 1.93, *P* = 0.09; the near position task: *t*_(7)_ = 2.27, *P* = 0.06]. A paired *t*-test demonstrated that the measured peak of the tangential hand velocities was not significantly different between the far and near position tasks [*t*_(7)_ = 1.10, *P* = 0.31]. However, in the measurement experiments the mean peak of the tangential hand velocity in the far position task (0.135 m/s) tended to be larger than that of the near position task (0.128 m/s). Our proposed method also showed the same tendency (the far position task: 0.124 m/s; the near position task: 0.119 m/s).

**Figure 6:**
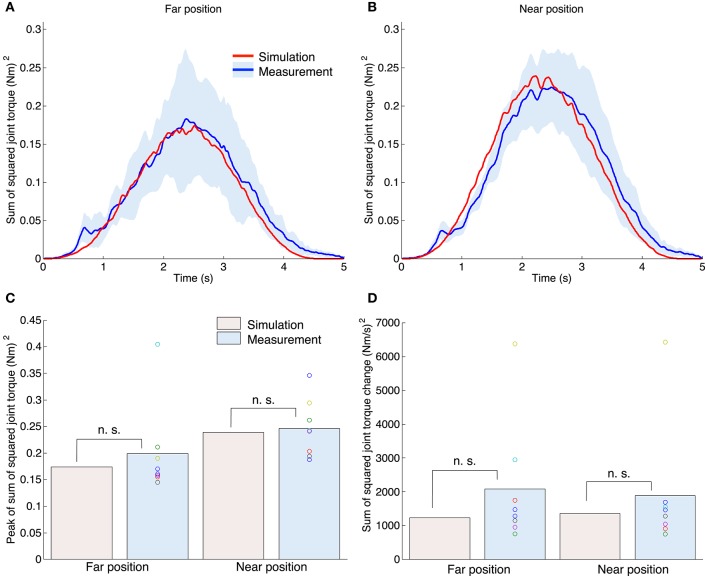
**Sum of the squared torques for all joints (shoulder, elbow, and wrist) for both the simulation and measurement experiments**. The upper left **(A)** and right **(B)** graphs correspond to the far position and near position tasks. The red and blue lines indicate the mean value profiles of the sum of the squared torque across all trials for the simulation experiments, and across all subjects for the measurement experiments. The blue area denotes the standard deviation across all subjects. **(C)** Peak of the sum of the squared torque for all experiments. The n. s. indicates not significant (one-sample *t*-test). **(D)** The mean value of the sum of the squared torque change for all experiments.

Figures 6A,B show the profiles of the sum of the squared torques for all joints. The red and blue solid lines indicate the results of the simulation and measurement experiments. The blue area denotes the standard deviation across all subjects. The sum of the squared torque was also bell-shaped while the profiles for the simulation and measurement experiments were similar. Figure 6C shows the peak of the sum of the squared joint torque for all experiments. A one-sample *t*-test between simulation and measurement results indicated that our proposed method could generate a similar peak to the measurement experiments [the far position task: *t*_(7)_ = 0.84, *P* = 0.43; the near position task: *t*_(7)_ = 0.38, *P* = 0.71]. A paired *t*-test demonstrated that the measured peak of the tangential hand velocities was not significantly different between the far and near position tasks [*t*_(7)_ = 1.37, *P* = 0.21]. However, for the measurement experiments the mean peak of the tangential hand velocity in the far position task [0.200 (Nm)^2^] tended to be smaller than that for the near position task [0.246 (Nm)^2^]. Our proposed method also showed the same tendency [far: 0.174 (Nm)^2^; near: 0.239 (Nm)^2^]. Figure 6D shows the mean sum of the squared torque change for all experiments. Figure 6D shows the sum of the squared joint torque change of all experiments. A one-sample *t*-test between the simulation and measurement results indicated that our proposed method could generate a peak value similar to the measurement experiments [the far position task: *t*_(7)_ = 1.30, *P* = 0.23; the near position task: *t*_(7)_ = 0.80, *P* = 0.45].

### Results of UCM analysis

**Figure 7:**
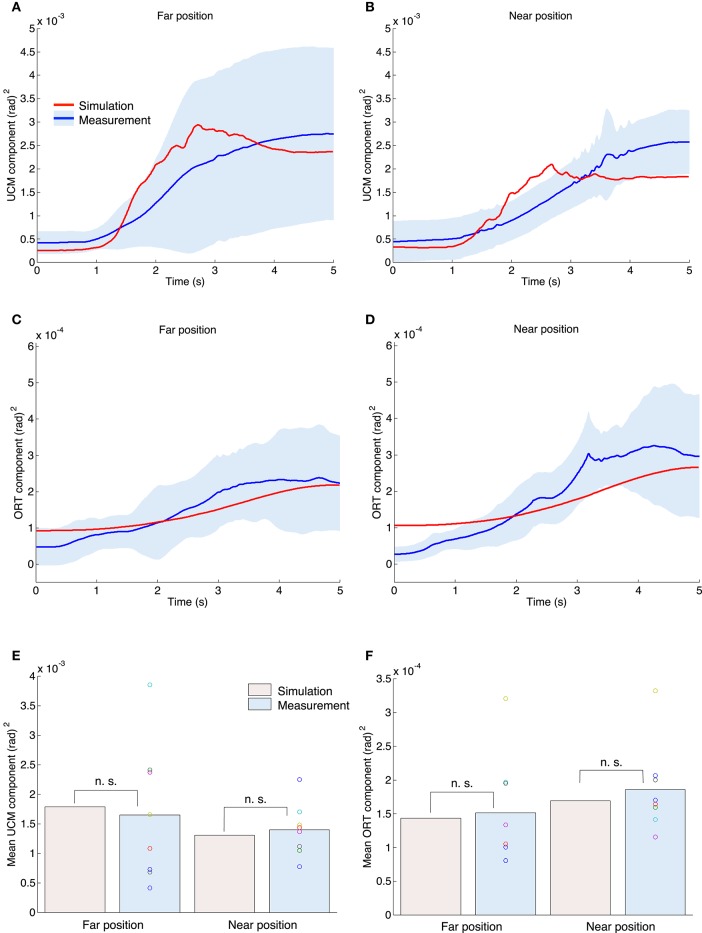
**UCM and ORT components for both the simulation and measurement experiments**. The upper left **(A,C)** and right **(B,D)** graphs correspond to the far position and near position tasks. The red and blue lines indicate the mean value profiles of the UCM and ORT components across all trials for the simulation experiments, and across all subjects for the measurement experiments. The blue area denotes the standard deviation across all subjects. **(E)** The mean value for the UCM components across the movement duration for all experiments. The n. s. indicates not significant (one-sample *t*-test). **(F)** The mean value for the ORT component.

Figures 7A–D show the waveforms of the UCM and ORT components. The upper and middle figures show results for the UCM and ORT components, respectively. The red and blue solid lines indicate the results of simulation and measurement experiments. The blue area denotes the standard deviation across all subjects. In both the simulation and measurement experiments, the mean waveforms of the UCM components were larger than those of the ORT components throughout the duration of movement, which indicates that the variance of joint angles was more varied across the UCM. Moreover, the UCM and ORT components gradually increased from the movement initiation to the end and our proposed method could generate same tendency. Figures 7E,F show the mean values for the UCM and ORT components for all experiments. A one-sample *t*-test between the simulation and measurement results indicated that our proposed method could generate a similar mean value for the UCM and ORT components from the measurement experiments [the UCM component in the far position task: *t*_(7)_ = −0.33, *P* = 0.75; in the near position task: *t*_(7)_ = 0.54, *P* = 0.60; the ORT component in the far position task: *t*_(7)_ = 0.28, *P* = 0.79; in the near position task: *t*_(7)_ = 0.73, *P* = 0.49].

A paired *t*-test demonstrated that the measured UCM and ORT components were not significantly different between the far and near position tasks [the UCM component: *t*_(7)_ = −0.61, *P* = 0.56; the ORT component: *t*_(7)_ = 1.66, *P* = 0.14]. However, for the measurement experiments the mean value for the UCM component in the far position task [0.0017 (rad)^2^] tended to be larger than that of the near position task [0.0014 (rad)^2^]. In contrast, the mean value for the ORT component in the far position task [1.52 × 10^−4^ (rad)^2^] tended to be smaller than that for the near position task [1.86 × 10^−4^ (rad)^2^]. Our proposed method also showed the same tendency, i.e., the UCM component in the far position task was 0.0018 (rad)^2^; the near position task was 0.0013 (rad)^2^; the ORT component in the far position task was 1.44 × 10^−4^ (rad)^2^; and the near position task was 1.69 × 10^−4^ (rad)^2^.

**Figure 8:**
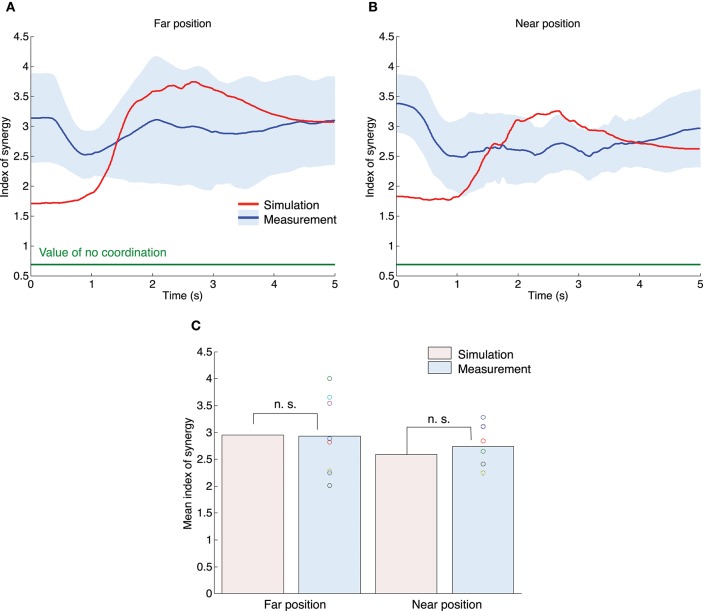
Index of synergy σ^*^ for the simulation and measurement experiments. The upper left **(A)** and right **(B)** graphs correspond to the far position and near position tasks. The red and blue lines indicate the mean value profiles of the index of synergy across all trials for the simulation experiments and across all subjects for the measurement experiments. The blue area denotes the standard deviation across all subjects. **(B)** The mean value for the index of synergy across the movement duration for all experiments. The n. s. indicates not significant (one-sample *t*-test).

Figures 8A,B show the profiles of index of synergy. The red and blue solid lines indicate the results of the simulation and measurement experiments. The blue area denotes the standard deviation across all subjects. The horizontal green line indicates the value of no coordination (*V*_UCM_ = *V*_ORT_). In both the simulation and measurement experiments, the index of synergy was larger than the value of no coordination, which indicates that the joint angles were well-coordinated. These results indicate that the UCM reference feedback control could generate a high index of synergy as well as the human subject. Figure 8C shows the mean value for the index of synergy for all experiments. A one-sample *t*-test between simulation and measurement results indicated that our proposed method could generate a similar mean value for the index of synergy as the measurement experiments [the far position task: *t*_(7)_ = −0.10, *P* = 0.92; the near position task: *t*_(7)_ = 1.03, *P* = 0.34]. A paired *t*-test demonstrated that the measured indices of synergy were not significantly different between the far and near position tasks [*t*_(7)_ = −0.94, *P* = 0.38]. However, for the measurement experiments the mean peak of the tangential hand velocity for the far position task (2.93) tended to be larger than that for the near position task (2.73). Our proposed method also showed the same tendency (the far position task was 2.96, and the near position task was 2.59).

## Discussion

The aim of this study was to propose a control method that directly used the UCM and generated smooth and effortless movements while quantitatively comparing against human arm movements. We proposed a UCM reference feedback control for coordinating a redundant joint arm. Our proposed algorithm generated the target UCM from the given target hand trajectory step by step. The target joint combination was then selected in that space so as to minimize the cost function with respect to the input joint torque and torque change. Results of the simulation and measurement experiments for the one-dimensional target-tracking task were quantitatively compared.

According to the statistical results, our proposed method quantitatively reproduced kinematic and dynamic properties such as end postures, the tangential velocity of the hand and the joint torque. In addition, good agreement of the joint coordination was confirmed by the UCM analysis. For the one-dimensional target-tracking task, subjects showed a larger UCM component than an ORT component (Figure 7) indicating that multi-joints were coordinated to control the hand. This result suggests that there is a coordinated control system in the human visuomotor system allowing for task-irrelevant variability. Our proposed method could quantitatively reproduce the mean value for the UCM and ORT components, the index of synergy and the tendency depending on the posture difference. These results indicate that the UCM reference feedback control can generate human-like joint coordination.

In addition, these results could not be reproduced by a control framework in which a unique pattern of joint trajectories was determined. In robotics, a pseudo-inverse matrix is often used to control a redundant joint to generate unique target joint trajectories throughout the movement duration beforehand, and each joint tracks the target (Whitney, 1969). If a human used such a control strategy in the one-dimensional target-tracking task, the person could repetitively generate unique target joint trajectories since the same target hand trajectory and the same initial position were provided. In such a case, if the joints were disturbed by some noise or perturbation, they would converge to unique target joint trajectories. Using the above strategy, however, the UCM component does not increase with a small ORT component, as shown in Figures 7A,B. Therefore, at least for the one-dimensional target-tracking task, it is suggested that the human uses a strategy such as the UCM reference feedback control rather than a strategy that preliminarily generates unique joint angle trajectories for the whole movement duration.

Our UCM reference feedback control references online whether the variance of joint angles affects task achievement. In human behavior studies, it was reported that the response to the task-relevant perturbation was enhanced and the task-irrelevant perturbation was inhibited (Diedrichsen, 2007; Franklin and Wolpert, 2008). Moreover, such a modulation of the response can be achieved only using somatosensory information without visual information (Dimitriou et al., 2012). Thus, the human visuomotor system may have a neural representation of the UCM, especially in the somatosensory feedback loop (lower loop in Figure 1C). For the target-tracking task, the target performance variable representing the one-dimensional hand position was given by visual information. Thus, the UCM was generated by using visual information and could be utilized in the somatosensory feedback loop. Examining whether the UCM is represented in the brain and how to represent the UCM are considered important future works.

As shown in Figure 4, the error in the workspace (*X*–*Y*) coordinates indicates that the UCM reference feedback control could more accurately track the target than human subjects. In contrast, the ORT components, which were properties in the joint space and would be affected by the variability of the initial postures, showed quantitatively similar results between the simulation and measurement experiments (Figure 7F). Since the delay of human visual feedback system is relatively long, the tracking error of the human subjects was large.

## Model assumptions

In this paper, our UCM reference feedback control employed two assumptions. First, the three variables in Figure 1C (i.e., *x*^*d*^, $\theta_{*}^{d}$, and **τ**) were exposed to noise. The noise of the input torque τrepresents the signal-dependent noise of a motor command (Harris and Wolpert, 1998). Additionally, the noise of the target hand position *x*^*d*^ represents the variability caused by target perception and localization with the human visual feedback system. Tseng et al. (2003) reported the relationship between the target size and the variability of joint angles in point-to-point reaching movements. They found that the variability of the joint angles increased with the size of the target. This suggests that the uncertainty of the perceived target affects the variability of joint angles. In addition, we assumed that the perception of the moving target would be more uncertain than the static target. Therefore, in our study such a relationship was represented by additive noise (Equation 16). The noise of the target joint combination $\theta_{*}^{d}$ represents the acquisition error of the UCM reference controller. As discussed above, we believe that the human CNS has an internal representation of information corresponding to the UCM, and we assume the acquisition uncertainty to be additive noise (Equation 17). The amplitudes of the noise *e*_τ_, *e*_*x*_, and *e*_θ_ affect the variability of the arm posture shown in Figure 3. The total variance of the joint is shown in Figure S1, and the profile of the input torque is shown in Figure 6.

Second, we used a cost function consisting of the input torque and torque change to select the unique joint combination for the target UCM. Alternatively, if we had used a cost function consisting of the minimum distance from the current joint combination to the target UCM in joint space it would be equivalent to using the pseudo-inverse matrix. In such a case, the required input torque would be much larger than the input torque as shown in Figure 6. Additionally, it would not be possible to reproduce the results of the measurement experiments. Therefore, a cost function based on the arm dynamics (e.g., torque and torque change) rather than a cost function related to the kinematics such as the pseudo-inverse matrix is required. Our cost function includes the torque change as a criterion for the smoothness of the human movement (Uno et al., 1989). Only the torque change term does not remain stationary since τ = 0 at the movement end; therefore, our cost function also includes the torque term (Equation 3) to generate effortless movements.

## Comparison with the other control method

When we used a cost function consisting of the sum of the squared norm of the joint angles instead of Equation (3), our proposed method was equivalent to a method in which the target joint angles were calculated for each time step using a pseudo-inverse matrix. Since the pseudo-inverse matrix does not consider the arm dynamics i.e., torque and torque change, the controller cannot smoothly, and effortlessly respond to noise and perturbation. Moreover, the pseudo-inverse matrix cannot reproduce a smooth bell-shape torque profile with motor noise as shown in Figure 6.

Figures 7, 8 show that our proposed method generates joint coordination while allowing variance that does not affect the task achievement. Such a “minimal intervention principle,” mentioned in the introduction, can also be realized within a framework of optimal feedback control (Todorov and Jordan, 2002b). The remarkable difference between a traditional optimal feedback control and our UCM reference feedback control is that the UCM is explicitly used in our method. In other words, our control scheme determines the optimal joint angle in UCM. This leads to the following advantages of the UCM reference feedback control. First, our proposed method can modularize an optimization process. In the traditional optimal feedback control framework, an input and output are simultaneously optimized using one cost function; e.g., *E* = ∫(***x***^*T*^***Wx***+**τ**^*T*^***Q*τ**) *dt*. Therefore, the generated input and output depend on the weights used in the cost function.

For the one-dimensional target-tracking task, the accuracy of target tracking and the amplitude of the input torque are a weight-dependent trade-off. It would be difficult for the traditional framework to optimally solve this weight-dependent trade-off, especially for a nonlinear system. Moreover, even if the trade-off problem is solved, new weights are required for the situation where the task conditions are changed (e.g., a large input torque may or may not be required). On the other hand, our UCM reference feedback control can divide the optimization process into a target joint combination decision (Figure 1C: UCM reference controller) and an input torque decision (Figure 1C: inverse dynamics). By this modularization, the weight-dependent trade-off can be avoided and new weights are not required even if the task condition is changed.

Second, our method can be applied to a nonlinear system such as the human arm while the tradition optimal feedback control cannot be directly applied. Todorov et al. applied the framework of optimal feedback control to a nonlinear system using a hierarchical control framework in which the controlled object was linearized around the virtual target trajectory resulting in the convergence of the cost function to an optimal solution through iterative computation (Todorov et al., 2005; Li and Todorov, 2007; Liu and Todorov, 2009). However, we demonstrated that UCM reference feedback control can be directly applied a nonlinear system without any iteration. Finally, our method can be applied to many types of redundant control reported in the previous studies of UCM analysis.

For example, previous studies examined the relationships between the joint angles and the center of mass (Freitas et al., 2010), individual finger forces and the total force (Kang et al., 2004), electromyograms and the center of pressure (Wang et al., 2006), electromyograms and the center of mass (Robert et al., 2008), and angular momenta of different components and whole-body angular momentum (Robert et al., 2009). In addition, tasks that were examined include postural tasks (Krishnamoorthy et al., 2003; dos Santos et al., 2007; Scholz et al., 2007; Robert et al., 2008; Wu et al., 2009), hopping (Auyang et al., 2009; Yen et al., 2009), pistol shooting (Scholz et al., 2000), walking (Black et al., 2007; Robert et al., 2009), writing by hand (Shim et al., 2010), and carrying a cup of water (Togo et al., 2012). Our UCM reference feedback control can be applied to all the above tasks by only providing the appropriate target of the performance variable and the cost function. We believe that UCM reference feedback control can be developed from joint coordination to comprehensive “synergy.”

The most remarkable difference between UCM reference feedback control and optimal feedback control is the process of optimization. In optimal feedback control, the optimal gain is calculated so as to minimize some cost function over the whole duration of the task. In contrast, our UCM reference feedback control model selects an optimal point in the target UCM at each time step. However, at the present stage of research it is difficult to conclude which model is more appropriate for human control model. UCM reference feedback control could generate human-like coordinated arm movements, but our results do not establish that the human CNS solves an optimization problem step by step. Instead, we conjecture that the human CNS generates an output close to the solution of an optimization problem based on the properties of the musculoskeletal system and a neural structure.

Extending the framework of UCM reference feedback control and improving physiological appropriateness, we would like to discuss the validity of the control model for humans in future work. As an example of an earlier study of human control model with redundancy, Martin et al. (2009) proposed a redundant control model with the human muscle model. They solved the redundancy problem by utilizing a pseudo-inverse matrix with a kinematic cost function, while our control model can deal with a cost function related to the joint torque for the redundancy. We would like to extend the UCM reference feedback control as a control model for humans by considering the muscle model, adding biological delay and including neural representation from a neural network. In that case, the method for controlling redundant muscles is also an important and interesting problem. Recently, it was reported that human CNS controls redundant muscle through some hierarchical motor primitives (d'Avella et al., 2003; Bizzi and Cheung, 2013; d'Avella and Lacquaniti, 2013) rather than optimal control (de Rugy et al., 2012; Berger et al., 2013). In future work, we need to combine the “muscle synergy,” which is the motor primitive for redundant muscle; and the “motor synergy,” which is a neural mechanism for generating joint coordination.

## Future works

In this study, we proposed the UCM reference feedback control to reproduce human multi-joint coordination. There are some other control techniques for optimally determining unique joint combination at each time step, e.g., inverse kinematics (Buss, 2004; Peters et al., 2007; Liu et al., 2015) and optimal feedback control (Toussaint, 2009; Rückert et al., 2013). We consider statistical comparisons of the performance of the related control methods as important future work. Moreover, our proposed method requires an internal model, specifically an inverse dynamics model. In future work, we would embed the learning schema of an internal model, e.g., the feedback error learning (Kawato et al., 1987).

## Author contributions

Conceived and designed the model and experiments: ST. Performed the experiments: ST. Analyzed the data: ST, TK, and YU. Wrote the paper: ST, TK, and YU. All authors gave final approval for publication.

## Funding

This research was supported by a Japan Society for the Promotion of Science (JSPS) Grant-in-Aid for JSPS Fellows 267576, and Grants-in-Aid for Scientific Research (B) No. 26289129 and 26280101.

### Conflict of interest statement

The authors declare that the research was conducted in the absence of any commercial or financial relationships that could be construed as a potential conflict of interest.

## Supplementary material

Figure S1 **Total variance for the simulation and measurement experiments**. The upper left **(A)** and right **(B)** graphs correspond to the far position and near position tasks. The red and blue lines indicate the mean values profiles for the total variance across all trials for the simulation experiments and across all subjects for the measurement experiments. The blue area denotes the standard deviation across all subjects. **(C)** The mean value for the total variance across the movement duration for all experiments. The n. s. indicates not significant (one-sample *t*-test).

## Appendix

### A. Calculation of cost function

Using constant terms ***B*** and ***F*** which are associated with the torque and torque change terms, the specific constant term ***A*** in Eq. (14) is as follows:

(A1) $$\begin{array}{l} A_{1}=B_{1}^{2}+B_{4}^{2}+B_{7}^{2}+F_{1}^{2}+F_{4}^{2}+F_{7}^{2}\\ A_{2}=2\left(B_{1}B_{3}+B_{4}B_{6}+B_{7}B_{9}+F_{1}F_{3}+F_{4}F_{6}+F_{7}F_{9}\right)\\ A_{3}=2\left(B_{1}B_{2}+B_{4}B_{5}+B_{7}B_{8}+F_{1}F_{2}+F_{4}F_{5}+F_{7}F_{8}\right)\\ A_{4}=2\left(B_{2}B_{3}+B_{5}B_{6}+B_{8}B_{9}+F_{2}F_{3}+F_{5}F_{6}+F_{8}F_{9}\right)\\ A_{5}=B_{2}^{2}+B_{5}^{2}+B_{8}^{2}+F_{2}^{2}+F_{5}^{2}+F_{8}^{2}\\ A_{6}=B_{3}^{2}+B_{6}^{2}+B_{9}^{2}+F_{3}^{2}+F_{6}^{2}+F_{9}^{2}. \end{array}$$

The specific constant terms B and C are as follows:

(A2) $$\begin{array}{l} B_{1}=\frac{W_{s}}{{\left(\Delta t\right)}^{2}}\left(-\frac{J_{2}^{†}}{J_{1}^{†}}M_{11}+M_{12}\right)\\ B_{2}=\frac{W_{s}}{{\left(\Delta t\right)}^{2}}\left(-\frac{J_{3}^{†}}{J_{1}^{†}}M_{11}+M_{13}\right)\\ B_{3}=\frac{W_{s}}{{\left(\Delta t\right)}^{2}}\left(-M_{12}\theta_{e}^{†}-M_{13}\theta_{w}^{†}-\frac{x_{d}-x^{†}+J_{2}^{†}\theta_{e}^{†}+J_{3}^{†}\theta_{w}^{†}}{J_{1}^{†}}M_{11}\right)\\ B_{4}=\frac{W_{e}}{{\left(\Delta t\right)}^{2}}\left(-\frac{J_{2}^{†}}{J_{1}^{†}}M_{21}+M_{22}\right)\\ B_{5}=\frac{W_{e}}{{\left(\Delta t\right)}^{2}}\left(-\frac{J_{3}^{†}}{J_{1}^{†}}M_{21}+M_{23}\right)\\ B_{6}=\frac{W_{e}}{{\left(\Delta t\right)}^{2}}\left(-M_{22}\theta_{e}^{†}-M_{23}\theta_{w}^{†}-\frac{x_{d}-x^{†}+J_{2}^{†}\theta_{e}^{†}+J_{3}^{†}\theta_{w}^{†}}{J_{1}^{†}}M_{21}\right)\\ B_{7}=\frac{W_{w}}{{\left(\Delta t\right)}^{2}}\left(-\frac{J_{2}^{†}}{J_{1}^{†}}M_{31}+M_{32}\right)\\ B_{8}=\frac{W_{w}}{{\left(\Delta t\right)}^{2}}\left(-\frac{J_{3}^{†}}{J_{1}^{†}}M_{31}+M_{33}\right)\\ B_{9}=\frac{W_{w}}{{\left(\Delta t\right)}^{2}}\left(-M_{32}\theta_{e}^{†}-M_{33}\theta_{w}^{†}-\frac{x_{d}-x^{†}+J_{2}^{†}\theta_{e}^{†}+J_{3}^{†}\theta_{w}^{†}}{J_{1}^{†}}M_{31}\right) \end{array}$$

(A3) $$\begin{array}{l} F_{1}=\frac{Q_{s}}{{\left(\Delta t\right)}^{3}}\left(-\frac{J_{2}^{‡}}{J_{1}^{‡}}M_{11}+M_{12}\right)\\ F_{2}=\frac{Q_{s}}{{\left(\Delta t\right)}^{3}}\left(-\frac{J_{3}^{‡}}{J_{1}^{‡}}M_{11}+M_{13}\right)\\ F_{3}=\frac{Q_{s}}{{\left(\Delta t\right)}^{3}}\left(-M_{12}\theta_{e}^{‡}-M_{13}\theta_{w}^{‡}-\frac{x_{d}-x^{‡}+J_{2}^{‡}\theta_{e}^{‡}+J_{3}^{‡}\theta_{w}^{‡}}{J_{1}^{‡}}M_{11}\right)\\ F_{4}=\frac{Q_{e}}{{\left(\Delta t\right)}^{3}}\left(-\frac{J_{2}^{‡}}{J_{1}^{‡}}M_{21}+M_{22}\right)\\ F_{5}=\frac{Q_{e}}{{\left(\Delta t\right)}^{3}}\left(-\frac{J_{3}^{‡}}{J_{1}^{‡}}M_{21}+M_{23}\right)\\ F_{6}=\frac{Q_{e}}{{\left(\Delta t\right)}^{3}}\left(-M_{22}\theta_{e}^{‡}-M_{23}\theta_{w}^{‡}-\frac{x_{d}-x^{‡}+J_{2}^{‡}\theta_{e}^{‡}+J_{3}^{‡}\theta_{w}^{‡}}{J_{1}^{‡}}M_{21}\right)\\ F_{7}=\frac{Q_{w}}{{\left(\Delta t\right)}^{3}}\left(-\frac{J_{2}^{‡}}{J_{1}^{‡}}M_{31}+M_{32}\right)\\ F_{8}=\frac{Q_{w}}{{\left(\Delta t\right)}^{3}}\left(-\frac{J_{3}^{‡}}{J_{1}^{‡}}M_{31}+M_{33}\right)\\ F_{9}=\frac{Q_{w}}{{\left(\Delta t\right)}^{3}}\left(-M_{32}\theta_{e}^{‡}-M_{33}\theta_{w}^{‡}-\frac{x_{d}-x^{‡}+J_{2}^{‡}\theta_{e}^{‡}+J_{3}^{‡}\theta_{w}^{‡}}{J_{1}^{‡}}M_{31}\right). \end{array}$$

The specific components of the inertia matrix ***M*** and Jacobian ***J*** are as follows:

(A4) $$\begin{array}{l} M_{11}=m_{1}l_{g1}^{2}+I_{1}+m_{2}\left(l_{1}^{2}+l_{g2}^{2}+2l_{1}l_{g2}\cos\theta_{e}\right)+I_{2}\\ \;+m_{3}(l_{1}^{2}+l_{2}^{2}+l_{g3}^{2}+2l_{1}l_{2}\cos\theta_{e}+2l_{1}l_{g3}\cos(\theta_{e}+\theta_{w})\\ \;+2l_{2}l_{g3}\cos\theta_{w})+I_{3}\\ M_{12}=M_{21}=m_{2}\left(l_{g2}^{2}+l_{1}l_{g2}\cos\theta_{e}\right)+I_{2}\\ \;+m_{3}(l_{2}^{2}+l_{g3}^{2}+l_{1}l_{2}\cos\theta_{e}+l_{1}l_{g3}\cos(\theta_{e}+\theta_{w})\\ \;+2l_{2}l_{g3}\cos\theta_{w})+I_{3}\\ M_{13}=M_{31}=m_{3}\left(l_{g3}^{2}+l_{1}l_{g3}\text{cos}(\theta_{e}+\theta_{w})+l_{2}l_{g3}\cos\theta_{w}\right)+I_{3}\\ M_{22}=m_{2}l_{g2}^{2}+I_{2}+m_{3}\left(l_{2}^{2}+l_{g3}^{2}+2l_{2}l_{g3}\cos\theta_{w}\right)+I_{3}\\ M_{23}=M_{32}=m_{3}\left(l_{g3}^{2}+l_{2}l_{g3}\cos\theta_{w}\right)+I_{3} \end{array}$$

(A5) $$\begin{array}{l} M_{33}=m_{3}l_{g3}^{2}+I_{3},\\ J_{1}^{†}=J_{2}^{†}-l_{1}\sin\theta_{s}^{†} \end{array}$$

(A6) $$\begin{array}{l} J_{2}^{†}=J_{3}^{†}-l_{2}\sin\left(\theta_{s}^{†}+\theta_{e}^{†}\right)\\ J_{3}^{†}=-l_{3}\sin\left(\theta_{s}^{†}+\theta_{e}^{†}+\theta_{w}^{†}\right),\\ J_{1}^{‡}=J_{2}^{‡}-l_{1}\sin\theta_{s}^{‡}\\ J_{2}^{‡}=J_{3}^{‡}-l_{2}\sin\left(\theta_{s}^{‡}+\theta_{e}^{‡}\right)\\ J_{3}^{‡}=-l_{3}\sin\left(\theta_{s}^{‡}+\theta_{e}^{‡}+\theta_{w}^{‡}\right). \end{array}$$

Using the constant term A, the extreme values $\theta_{s}^{d*}$ and $\theta_{s}^{d*}$ are represented as follows:

(A7) $$\begin{array}{l} \theta_{e}^{d*}=\frac{A_{3}A_{4}-{2A}_{2}A_{5}}{4A_{1}A_{5}-A_{3}^{2}}\theta_{e}^{d*}=\frac{A_{2}A_{3}-{2A}_{1}A_{4}}{4A_{1}A_{5}-A_{3}^{2}}. \end{array}$$

### B. Supplementary figures

Figures S1A and S1B show profiles for the total variance. The red and blue solid lines indicate the results of the simulation and measurement experiments. The blue area denotes the standard deviation across all subjects. In both the simulation and measurement experiments, the total variance gradually increased from the movement start to end. Our proposed method could generate same tendency. Figure S1C shows the mean total variance for all experiments. A one-sample *t*-test indicated that our proposed method could generate a similar mean value for the total variance to the measurement experiments [the far position task: *t*_(7)_ = −0.32, *P* = 0.76; the near position task: *t*_(7)_ = 0.59, *P* = 0.57]. A paired *t*-test demonstrated that the mean total variances were not significantly different between the far and near position tasks [*t*_(7)_ = −0.56, *P* = 0.60]. However, for the measurement experiments the mean total variance for the far position task [0.0012 (rad)^2^] tended to be larger than that in the near position task [9.95 × 10^−4^ (rad)^2^]. Our proposed method also showed the same tendency [the far position task: 0.0012 (rad)^2^; the near position task: 9.32 × 10^−4^ (rad)^2^].
